# 4,4′-Diaminodiphenyl Sulfone (DDS) as an Inflammasome Competitor

**DOI:** 10.3390/ijms21175953

**Published:** 2020-08-19

**Authors:** Jong-hoon Lee, Ha Kyeu An, Mun-Gi Sohn, Paul Kivela, Sangsuk Oh

**Affiliations:** 1 Science and Research Center, Seoul National University College of Medicine, Seoul 03080, Korea; science@research.re.kr; 2 Department of Neurology, Sorokdo National Hospital, Jeollanam-do 59562, Korea; hakyeu0714@gmail.com; 3 Department of Food Science, KyungHee University College of Life Science, Seoul 17104, Korea; mgsohn@khu.ac.kr; 4 Department of Emergency Medicine, University of Alabama at Birmingham, Birmingham, AL 35233, USA; 5 Department of Food Engineering, Food Safety Laboratory, Memory Unit, Ewha Womans University, Seoul 03670, Korea

**Keywords:** AD, Alzheimer’s disease, DDS, 4,4′-diaminodiphenyl sulfone (dapsone), LL, lepromatous leprosy (also known as Hansen’s disease), MADDS, monoacetyldapsone, NLRP3, NACHT, LRR and PYD domains-containing protein 3, MPO, myeloperoxidase, TLR, toll-like receptor

## Abstract

The aim of this study is to examine the use of an inflammasome competitor as a preventative agent. Coronaviruses have zoonotic potential due to the adaptability of their S protein to bind receptors of other species, most notably demonstrated by SARS-CoV. The binding of SARS-CoV-2 to TLR (Toll-like receptor) causes the release of pro-IL-1β, which is cleaved by caspase-1, followed by the formation and activation of the inflammasome, which is a mediator of lung inflammation, fever, and fibrosis. The NLRP3 (NACHT, LRR and PYD domains-containing protein 3) inflammasome is implicated in a variety of human diseases including Alzheimer’s disease (AD), prion diseases, type 2 diabetes, and numerous infectious diseases. By examining the use of 4,4′-diaminodiphenyl sulfone (DDS) in the treatment of patients with Hansen’s disease, also diagnosed as Alzheimer’s disease, this study demonstrates the diverse mechanisms involved in the activation of inflammasomes. TLRs, due to genetic polymorphisms, can alter the immune response to a wide variety of microbial ligands, including viruses. In particular, TLR2Arg^677^Trp was reported to be exclusively present in Korean patients with lepromatous leprosy (LL). Previously, mutation of the intracellular domain of TLR2 has demonstrated its role in determining the susceptibility to LL, though LL was successfully treated using a combination of DDS with rifampicin and clofazimine. Of the three tested antibiotics, DDS was effective in the molecular regulation of NLRP3 inflammasome activators that are important in mild cognitive impairment (MCI), Parkinson’s disease (PD), and AD. The specific targeting of NLRP3 itself or up-/downstream factors of the NLRP3 inflammasome by DDS may be responsible for its observed preventive effects, functioning as a competitor.

## 1. Introduction

SARS-CoV-2 is an acute disease from which sufferers generally recover, though it can also be deadly, with the fatality rate estimated at 2% [1]. Severe disease onset might result in death due to massive alveolar damage and progressive respiratory failure. Most patients with SARS-CoV-2 have a mild disease course; however, approximately 20% develop severe disease, and a high mortality rate is associated with older age and immunosuppression.

Coronaviruses infect a wide range of mammals and birds. Their tropism is primarily determined by the ability of the spike (S) entry protein to bind to a cell surface receptor. Coronaviruses have zoonotic potential due to the adaptability of their S protein to receptors of other species, most notably demonstrated by SARS-CoV [2]. MERS (Middle East Respiratory Syndrome) -CoV binds to dipeptidyl peptidase 4 (DPP4; also known as CD26) receptors that are primarily in the lower respiratory tract but also distributed in other tissues [3].

It is important to investigate whether other host genetic factors could influence susceptibility to SARS-CoV infection and its subsequent clinical course. The innate immune system plays a role in limiting infectious challenge in the early stages after exposure during the lag time required to initiate long-lasting adaptive immune system [4]. When SARS-CoV-2 infects the upper and lower respiratory tract, it can cause mild or highly acute respiratory syndrome with the subsequent release of proinflammatory cytokines, including interleukin (IL)-1β and IL-6. The binding of SARS-CoV-2 to the Toll-like receptor (TLR) causes the release of pro-IL-1β, which is cleaved by caspase-1, followed by inflammasome activation and the production of active, mature IL-1β, which is a mediator of lung inflammation, fever, and fibrosis. The suppression of proinflammatory IL-1 family members and IL-6 has been shown to have a therapeutic effect in many inflammatory diseases, including viral infections [5].

After RNA virus infection, NLRP3 inflammasomes were activated by mitochondrial protein mitofusin 2 [6]. The mitochondrial antiviral signaling protein (MAVS) associates with NLRP3 and regulates its inflammasome activity [7]. NLRP3 is an intracellular sensor that detects a broad range of microbial motifs, endogenous danger signals, and environmental irritants, resulting in the formation and activation of the NLRP3 inflammasome. Assembly of the NLRP3 inflammasome leads to caspase-1-dependent release of the pro inflammatory cytokines IL-1β and IL-18, as well as to gasdermin D-mediated pyroptotic cell death. The dysfunction of NLRP3 (which encodes NOD-, LRR-, and pyrin domain-containing protein 3) inflammasome activation is implicated in a variety of human diseases, including Alzheimer’s disease (AD), prion diseases, type 2 diabetes, and numerous infectious diseases [8]. The activation of cytokines or pathogen-associated molecular patterns (PAMPs) leads to the transcriptional upregulation of canonical and noncanonical inflammasome components [9]. 

Multiple downstream events may be activated by a variety of upstream signaling PAMPs or damage-associated molecular patterns (DAMPs), such as particulates, crystals, or ATP (Adenosine triphosphate). These include K+ efflux, Ca+ flux, lysosomal disruption, mitochondrial reactive oxygen species (mtROS) production, the relocalization of cardiolipin to the outer mitochondrial membrane, and the release of oxidized mitochondrial DNA (Ox-mtDNA) followed by Cl^−^ efflux. RNA viruses activate NLRP3 through MAVS on the mitochondrial outer membrane. Formation of the inflammasome activates caspase-1 which, in turn, cleaves pro-IL-1β and pro-IL-18 [9].

At present, the factors that ultimately determine NLRP3 inflammasome activation and common signaling pathways targeted by NLRP3 inflammasome activation remain unknown. The specific targeting of NLRP3 itself, and not other components (NEK7 (NIMA-related kinase 7), ASC (apoptosis-associated speck-like protein containing CARD), caspase-1 (Interleukin-1 converting enzyme), or IL-1β (leukocytic pyrogen)) or the up-/downstream factors of the NLRP3 inflammasome, may produce therapeutic effects. In this study, we developed 4,4′-diaminodiphenyl sulfone (DDS) as an Inflammasome Competitor. 

DDS has been used as an oral drug since 1949 [10]. Initially, it was approved for leprosy, for which it is still frequently used. In addition to its antimicrobial effects, DDS is a potent anti-inflammatory agent with high effectiveness in dermatitis herpetiformis and a wide variety of other inflammatory dermatological conditions [11]. Although DDS is generally well tolerated and suitable for long-term treatment, adverse drug reactions may occur. Obligatory (dose-dependent) adverse drug reactions include hemolytic anemia and methemoglobinemia [12]. Important, less well-known, potentially fatal adverse drug reactions with unknown pathomechanisms are hypersensitivity reactions to DDS, such as dapsone hypersensitivity syndrome [13]. Clinicians should be aware of hypersensitivity reactions to DDS [14].

## 2. Materials and Methods

### 2.1. Respiratory Tract Specimens and Symptoms of COVID-19

Upper respiratory tract (URT) specimens were collected from confirmed patients every day after the diagnosis of SARS-CoV-2 infection and sent to the Korea Center for Disease Control (CDC) for follow-up tests and culture. Real-time reverse transcriptase polymerase chain reaction was used to detect SARS-CoV-2. RNA was extracted from clinical samples followed by instruction manual from the company [15]. All specimens were handled in a biosafety cabinet according to laboratory biosafety guidelines of Korea CDC for SARS-CoV-2 by medical staff (including volunteers) (Supplement. 1). Specimens were also used to diagnose the presence or absence of COVID-19; outcomes from these clinical assessments are not included here and are reserved for future cohort studies. Clinical manifestations of COVID-19 infection include fever, fatigue, myalgia, headache, diarrhea, dry cough, immune dysregulation, vasculitis, vessel thrombosis, hypercoagulable state and dyspnea that may lead to acute respiratory distress syndrome and death [16]. We will find the most similar drugs that cause clinical manifestations of COVID-19 infection in humans and suggest a mechanism of action.

### 2.2. The Topological Properties of DDS Provide Diverse Clues

The molecular properties of DDS, including electron density and its Laplacian delocalization index, have been elucidated to shed light on the chemical bonding and atomic and molecular details [17]. The function of electronical localization has been used to visualize and deduce information on the lone pair and the subshells of the C atom. The theoretical charge density and topological properties of the gas phase of the molecule have been applied [18].

### 2.3. The Longevity of Leprosy-Affected Males in Spite of Several Pandemic Viral Diseases

Most data were obtained from the Korea Hansen (Leprosy) Welfare Association (KHWA) and Sorokdo National Hospital for the leprosy affected population. The KHWA is a government-sponsored organization with the goal of improving welfare systems for leprosy-affected persons in Korea.

Leprosy-affected male (among 14,686 persons, as of 1 January 2008), showed higher longevity index values, longer life expectancies, and lower age-specific death rates than their male counterparts in the comparison groups. However, these trends were not found in leprosy-affected females. Leprosy infection may have led this population to maintain healthy lifestyles and beliefs, particularly for males, more than all Koreans or Koreans of a low socioeconomic status [19]. 

We also reviewed the death certificate data of 1359 leprosy-affected people from 2010 to 2013. The all-cause mortality of 1359 (2010–2013) was significantly lower than that in the general population, corresponding to an SMR (Standard Mortality Ratio) of 0.84 (95% CI (Confidential Interval) 0.79–0.88). Malignancy (due to liver cancer) had the highest standardized mortality, with 130.9 deaths per 100,000 persons (40.0 deaths), followed by cardiovascular diseases (CVDs; 85.5 deaths) and respiratory diseases (38.2 deaths) [20]. 

Since 1996, the National Health Insurance Service (NHIS) in Korea has maintained medical records with the International Classification of Diseases (ICD) -9 code. Moreover, with Electronic Data Interchange (EDI), the Sorokdo National Hospital has archived their medical records. We connected to the EDI database of the Sorokdo National Hospital, archived from January 2005 to June 2020, and searched using the ICD-9 and 10 codes. Using this available archive, Hansen’s patients were evaluated for prescription DDS or not at Sorokdo National Hospital, dedicated to treating Hansen’s patients. We analyzed infectious respiratory diseases of Hansen’s subjects according to the Official Information Disclosure Act in Korea. Medical data on the correlation between DDS and infectious respiratory diseases were then analyzed using the software of Object—Relational DBMS and SPSS.

### 2.4. Cohort Study for Treatment of Alzheimer’s Disease with 4,4′-Diaminodiphenylsulfone 

This study used medical records from 2007 to 2020 and stages 2 to 6 (2007–2018.6) and 6 to 3 (2018.6–2019.1) of numeric clinical staging (NCS) according to the 2018 NIA-AA Research Framework. The medical records of the Seoul study originated from the published research article “Recovery of Dementia Syndrome following Treatment of Brain Inflammation” [21]. The Seoul study is a prospective cohort based on brain inflammation treatment of MCI and AD-affected elderly individuals in Korea.

According to the diagnostic criteria of AD, there are neuroinflammatory regions that are excluded from the treatment of AD. Numeric clinical staging (NCS) was recorded, including side effects of the most commonly used AADs and symptoms in the neuroinflammatory area. In addition, the treatment process was classified by managing the AD symptoms and neuropsychiatric (NP) symptoms caused by AADs with a new biomarker (D). By introducing a new biomarker (D), the progression of AD was newly monitored through NCS staging.

## 3. Results

### 3.1. DDS Has the Clinical Manifestations of COVID-19 Infection in Humans

We were particularly drawn to their description of COVID-19 patients presenting with multi-organ symptoms mimicking internal, surgical, dermatological, immunological and renal diseases, specifically a ‘DDS-like clinical presentation’. We compared each of the effects on humans and we found that SARS-CoV-2 and DDS must be very similar substances to humans. (Table 1)

### 3.2. DDS Has Nucleophilic Sites for Ubiquitination

The electrostatic potential visualizes the positive and negative electrostatic potential regions which are susceptible to nucleophilic and electrophilic attack [38]. The symmetry level was determined by DDS molecular charge analysis. Density functional calculations show that the symmetric conformational isomer has lower energy than the asymmetric conformational isomer. The aniline ring is the nucleophilic moiety conferring possible biological properties via a redox mechanism, mainly electron transfer or oxidation for DDS‒NHOH formation [39]. 

**Figure 1:**
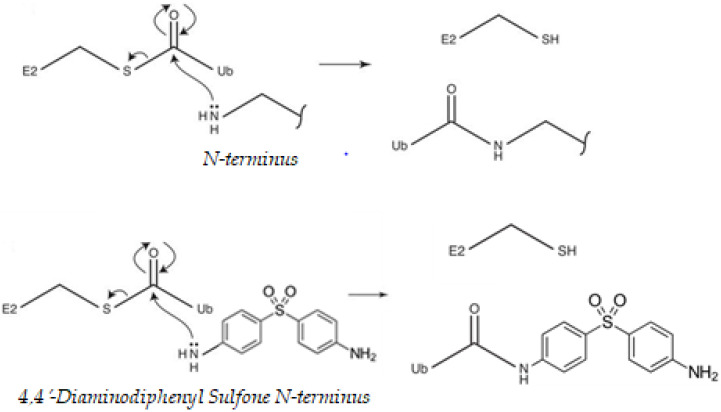
Nucleophilic properties of, 4′-diaminodiphenyl sulfone (DDS). Proteins contain many nucleophilic sites capable of attacking a ubiquitin (Ub)-conjugating enzyme (E2)–Ub thioester linkage and undergoing ubiquitination. The best-described sites are the amine-containing internal lysine residues and the free amine of the N-terminus of the polypeptide backbone. Ub is activated by an Ub-activating (E1) enzyme, using energy from ATP hydrolysis, and passed to an Ub-conjugating (E2) enzyme. Ub can then be passed to a substrate protein, specified by the distinct E3 ligase that binds both the substrate and the E2. DDS can compete with the ubiquitination cascade. Cysteine thiols and hydroxyls on serines, threonines, leucines, and tyrosines could also potentially be ubiquitinated by an identical mechanism [41,43,44].

That cysteine residues might undergo modification by ubiquitination may seem counterintuitive at first, as thioester linkages can be broken under reducing conditions and the intracellular environment is often viewed as “reducing” [40]. At each stage of the ubiquitination process, before loading of ubiquitin (Ub) onto the substrate, the Ub-activating (E1)/Ub-conjugating (E2)/E3 ligase enzymes are all able to carry ubiquitin via a thioester linkage, which is used to allow energetically favorable attack of the substrate nucleophile. Lysine/N-terminal amines, cysteine thiols or serine/threonine hydroxyls, attacks the electrophile thioester carbonyl of an E2–ubiquitin (Ub) conjugate. This results in a Ub–substrate conjugate linked by an isopeptide (lysine), peptide (N-terminal), thioester (cysteine) or hydroxyester bond (serine/threonine), respectively [41]. DDS, in particular, has nucleophilic sites for ubiquitination. Proteins contain many nucleophilic sites capable of attacking an E2–Ub thioester linkage and undergoing ubiquitination. The free amine of the N-terminus of DDS could also potentially be ubiquitinated by an identical pathway (Figure 1). The extent to which transmission is operative in the sulfone is dependent on the precise nature of the para substituents, in particular whether they are donors or acceptors and the manner in which they interact with the remainder of the molecule [42]. The results show the influence of amine.

### 3.3. DDS Should Be Effective for Molecular Regulation of Inflammasome Activators

Both SARS-CoV and SARS-CoV-2 are pH dependent and require acidification of endosomes [45,46] as well as lysosomes [47] to infect the cells. DDS has multiple activities. Fist, DDS blocks myeloperoxidase (MPO), which hampers the low-pH-dependent steps of viral replication, including fusion and uncoating. Myeloperoxidase is a kind of oxidoreductase that catalyzes the chemical reaction of the following reaction: H_2_O_2_ + Cl^−^ = H_2_O + OCl^−^. DDS binds to myeloperoxidase and regulates the production of hypochlorite, thereby reducing the inflammatory response of cells [48,49,50]. This may explain vulnerability in the elderly, diabetics, and obese patients.

TLRs are part of the innate immune system. TLR2 recognizes lipoproteins and TLR4 lipopolysaccharides (LPS). TLR activation occurs through receptor dimerization [51]. The mutation TLR2Arg^677^Trp [52] was only involved in the development of lepromatous leprosy (LL), and not tuberculoid leprosy (TL) or controls. Korean patients with almost lepromatous leprosy have been successfully treated in spite of gene mutations related with drug resistance in Mycobacterium leprae [51]. Since LL and TL have been successfully treated by DDS, the mutation (polymorphism) in the intracellular domain of TLR2 does not play a role in exacerbating LL. DDS act in the cell, like inflammasome.

**Figure 2:**
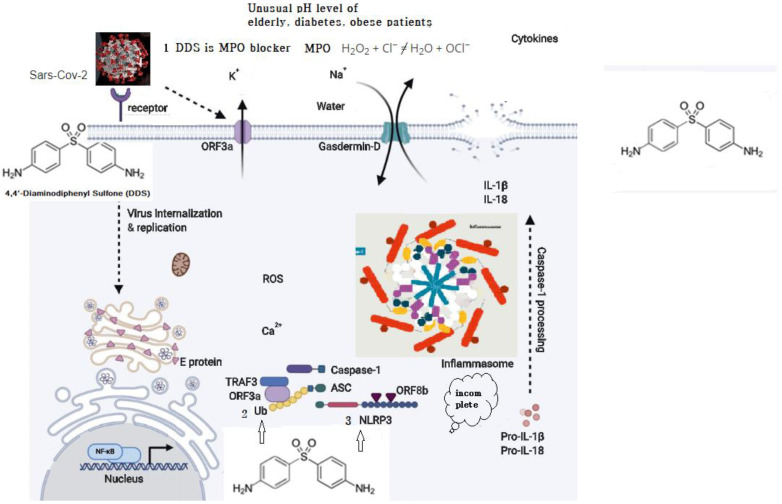
Possible schematic diagram: blocking of SARS-CoV-2 by DDS.

SARS-CoV which caused the severe acute respiratory syndrome global epidemic between 2002 and 2003 was shown to express at least three proteins that activate the NLRP3 inflammasome: envelope (E), ORF (open reading frame) 3a, and ORF8b. E protein and ORF3a are able to stimulate NF-Kb (a protein complex that controls transcription of DNA, cytokine production and cell survival) signaling to drive the transcription of inflammatory cytokines and chemokines, including IL-1b, IL-18, and IL-8, and prime NLRP3 expression to its functional level. ORF3a also activates the NLRP3 inflammasome by promoting tumor necrosis factor alpha (TNFR)-associated factor 3 (TRAF3)–mediated ubiquitination of ASC [53]. ORF8b activates NLRP3 through direct interaction with the leucine-rich repeat domain of NLRP3 [54]. SARS-CoV-2 shares ∼79% overall genetic similarity with SARS-CoV and the amino acid sequences of SARS-CoV-2 [55,56]. It is likely that SARS-CoV-2 could also activate the NLRP3 inflammasome. Nucleophilic properties of DDS compete with ORFb in the leucine-rich repeat domain of NLRP3. (Figure 2).

1. Myeloperoxidase is a kind of oxidoreductase that catalyzes the chemical reaction of the following reaction: H_2_O_2_ + Cl^−^ = H_2_O + OCl^−^. DDS binds to myeloperoxidase and regulates the production of hypochlorite, thereby reducing the inflammatory response of cells.
2. Nucleophilic properties of DDS compete with Ub.
3. Nucleophilic properties of DDS compete with NLRP3. ORF8b activates NLRP3 through direct interaction of the leucine-rich repeat domain of NLRP3.

Molecular docking provided detailed information about the formation of hydrogen bonding in the DDS–DNA complex. This in silico study further revealed that DDS binds to the AT-rich region of the minor groove of DNA, having a relative binding energy of −6.22 kcal mol^−1^ [57]. The currently available evidence indicates that DDS can exert various anti-inflammatory effects by inhibiting the generation of toxic free radicals, myeloperoxidase-mediated halogenation that converts H_2_O_2_ to HOCl^−^, leukocyte chemotaxis, the production of tumor necrosis factor, and other anti-inflammatory molecules [58].

### 3.4. General Profiles of Leprosy-Affected Elderly in Korea

The population of Korea has suffered severely from SARS-CoV (2002), Influenza A virus subtype H1N1 (2009), MERS (2015), and SARS-CoV-2 (2020). However, the Korean Hansen Welfare Association reported that Hansen’s patients in Korea had no reports of any outbreaks of respiratory infectious diseases between 2002 and 2019. 

The recurrence of Hansen’s disease has been reported through regular medical examinations and diagnosis. (Table 2) 

Korea Centers for Disease Control and Prevention (KCDC) is supplying DDS to Hansen’s patients. KCDC reported that DDS was supplied free for all (DDS 100 mg–1000 tablet/bottle to 3814 of 9134 Hansen’s patients). With a bottle, each of three people can take one tablet per day for one year.

**Figure 3:**
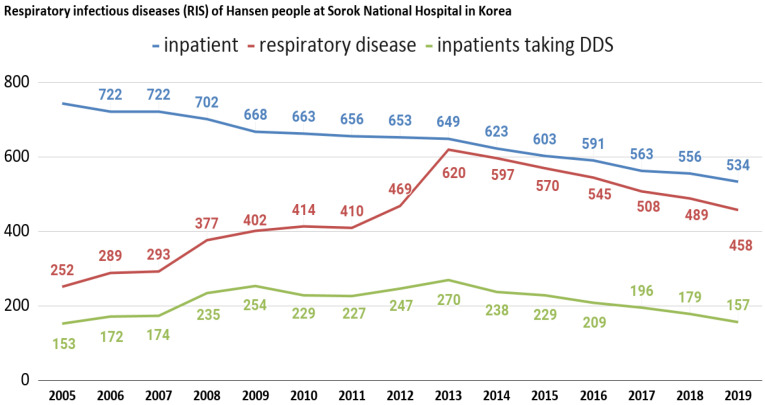
Respiratory infectious diseases (RIS) of Hansen’s patients at Sorokdo National Hospital in Korea. International Classification of Diseases (ICD-9, 10) of RIS are J00 (acute nasopharyngitis), J01 (acute sinusitis), J01.8 (other acute sinusitis), J02 (acute pharyngitis), J02.9 (acute pharyngitis, unspecified), J03 (acute tonsillitis), J03.0 (streptococcal tonsillitis), J03.9 (acute tonsillitis, unspecified), J04.0 (acute laryngitis), J06.0 (acute laryngopharyngitis), J06.9 (acute upper respiratory infection, unspecified), J09 (influenza due to identified zoonotic or pandemic influenza virus), J10.8 (influenza with other manifestations, seasonal influenza virus identified), J12.9 (viral pneumonia, unspecified), J15.8 (other bacterial pneumonia), J15.9 (bacterial pneumonia, unspecified), J18.0 (bronchopneumonia, unspecified), J18.9 (pneumonia, unspecified), J20.9 (acute bronchitis, unspecified), J31 (chronic rhinitis, nasopharyngitis and pharyngitis), J31.0 (chronic rhinitis), J31.1 (chronic nasopharyngitis), J31.2 (chronic pharyngitis), J32 (chronic sinusitis), J32.4 (chronic pansinusitis), J32.8 (other chronic sinusitis), J35. 0 (chronic tonsillitis), J37.0 (chronic laryngitis). This is a graph that can guess the characteristics of DDS as a competitor when comparing the proportion of RIS with those who have been prescribed DDS. After President Dae-Jung Kim came to power in 1998, Hansen’s patients were given freedom. Many were devout Christians, but as more and more became smokers, as in a typical rural village, RIS increased. Sorok-do became a tourist destination and travelers have come and gone freely, but SARS-CoV (2002), influenza A virus subtype H1N1 (2009), MERS (2015), and SARS-CoV-2 (2020) have not occurred.

We also analyzed infectious respiratory diseases among Hansen’s patients at Sorokdo National Hospital. The data shows that the patients of Sorokdo National Hospital are responsible for the development of respiratory infectious diseases according to the occurrence of SARS-CoV (2002), Influenza A virus subtype H1N1 (2009), MERS (2015), and SARS-CoV-2 (2020). There is no big change and no statistical correlation (Figure 3).

All patients have continued to take DDS after they were diagnosed. The proportion of the oldest demographic group (80 years and over) is still 38.5% (2019) higher than for all Koreans (Table 3).

The proportion of those aged 80 and over has been at least 50% higher for the leprosy-affected male population than for all male Koreans since 1995. The same is also shown for females, although the ratios are much smaller than those of males [19]. The Hongerwinter (hunger winter) began late in 1944 towards the end of the Second World War. Individuals who were prenatally exposed to famine during the Dutch Hunger Winter in 1944–45 had, six decades later, less DNA methylation of the imprinted IGF2 (Insulin-like growth factor 2) gene compared with their unexposed, same-sex siblings. Years afterward, the same individuals showed an abnormally elevated frequency of obesity, cardiac disease, and even schizophrenia, despite the lack of genetic history of these diseases. DNA methylation patterns in specific regions of the genome were altered in many of the Hongerwinter children [59,60]. The life-prolonging effect of caloric restriction in yeasts requires NPT1 and SIR2 genes, both of which relate to sensing energy status and silencing genes [61]. Leprosy-affected males had been isolated and managed to maintain healthy lifestyles and faith. They have been living freely since the 2000s. It can be assumed that one consistent reason for their long lives and the fact that they are free of infectious diseases is due to taking DDS regularly.

### 3.5. A Prospective Cohort Study for Neuroinflammation

DDS, among three antibiotics, appears to be effective in molecular regulation for the treatment of mild cognitive impairment (MCI), AD, and Parkinson’s disease (PD). A prospective cohort study reported an elderly patient who had MCI from February 2008 to January 2019. The patient took DDS 100 mg once a day from 2010 to 2015 for the treatment of MCI. In 2016, the production of DDS for the local pharmacy was ceased in Korea. In June 2018, the patient was then diagnosed with Alzheimer’s disease.

In this study, an anti-Alzheimer’s disease drug (AAD) was used as a new biomarker of NCS (D). During the periods of 2018.06.27–2018.10.01 and 2018.11.06–2018.11.21, the side effects caused by AAD occurred, and the patient was in NCS stage 6. We also examined other patients who had the same symptoms. In addition, from 2018.10.01 and 2018.11.22, after stopping the AAD, the disease changed to NCS stage 6 → 5. After neuroinflammation treatment, it changed again to NCS stage 5 → 3. 

DDS was re-administered to the patient from November 2018. The patient recovered from MCI and experienced improvements in their daily life owing to the treatment with DDS, which controls the inflammatory response in the brain, irrespective of whether proteins are deposited in neurons [37]. This indicates that DDS regulates inflammation in neurons. (Table 4)

Adult respiratory distress syndrome due to SARS-CoV-2 infection is associated with encephalopathy, agitation, confusion, and corticospinal tract signs. Two out of 13 patients who underwent a brain MRI (Magnetic Resonance Imaging) had single acute ischemic strokes. Data are lacking to determine which of these features were due to critical illness-related encephalopathy, cytokines, the effect or withdrawal of medication, and which features were specific to SARS-CoV-2 infection [35,36]. To date, clinical studies have shown that DDS can block disease progression due to critical illness (inflammation)-related encephalopathy or immune-related diseases [36,37,62,63]. 

We re-examined the function of hydrogen peroxidase in relation to hematocrit and microfibrin formation, which results in various cascade reactions, such as canonical–noncanonical ubiquitylation, NLRP3 inflammasome formation, Higgins’ cascade and strongly magnetic iron-rich nanoparticles, by RBC splitting. Through the same process, DDS can prevent the neuropsychiatric exacerbation of SARS-CoV-2 infection.

## 4. Discussion

### 4.1. This Special Issue on “Myeloperoxidase”

Myeloperoxidase (MPO) activity, a marker of inflammatory response, is increased 3.7-fold in ischemic animals vs. control rats, and this effect is antagonized by DDS treatment. Although apoptosis increased due to the effect of ischemia at both evaluation times, DDS antagonized that effect only 72 h after surgery. In all measured ischemia‒reperfusion end points, DDS treatment showed a remarkable ability to decrease markers of cell damage by antioxidant, anti-inflammatory, and anti-apoptotic effects [64,65]. DDS is effective in surgical stress induced by brain oxidative damage via downregulating nicotinamide adenine dinucleotide phosphate (NADPH) oxidase level in aged mice [65], propofol-induced cognitive alterations in aged rats [66], and an anti-apoptotic effect after spinal cord injury [67]. 

Oxidative stress markers as well as high concentrations of copper are found in the vicinity of amyloid β (Aβ) deposits in Alzheimer’s disease. The neurotoxicity of Aβ in brain cells has been linked to H_2_O_2_ generation by a study conducted in Seoul. Cu(II) and Fe(III) have been found in abnormally high concentrations in amyloid plaques (0.4 and 1 μM, respectively) and AD-affected neuropil [67]. Cu(II) markedly potentiates the neurotoxicity exhibited by Aβ in a cell culture. The potentiation of toxicity is greatest for Aβ1–42 > Aβ1–40 >> mouse/rat Aβ 1–40, corresponding to their relative capacities to reduce Cu(II) to Cu(I), form H_2_O_2_ in cell-free assays, and exhibit amyloid pathology. Since Cu(II)-glycine alone was not neurotoxic, these results strongly support the possibility that Cu(II) interaction modifies Aβ, leading to enhanced H_2_O_2_-mediated neurotoxicity. Copper, iron, and zinc may be important in exacerbating and perhaps facilitating Aβ-mediated oxidative damage in several diseases [67]. DDS is a therapeutic preventive substance in AD [68,69,70,71]. DDS (100 mg/day) and a placebo were orally administered once daily for 52 weeks in 201 patients with mild to moderate AD. At the end of treatment, there were no significant differences between DDS and the placebo in terms of cognitive or other measures of efficacy [72,73]. Those results are because patients with mild to moderate AD already have extensive neuronal death, and patients with MCI and early AD patients in the Seoul study were positive. MCI did not develop into AD for 10 years, and PD symptoms in the Seoul study became milder [37].

### 4.2. Molecular Regulation of Inflammasome Activators

The sulfur (S) atom exhibits a very high positive Bader atomic charge (2.36e) as it is attached with the two most electronegative oxygen (O) atoms (−1.27e) on both sides. The calculated angle for the plane O-S-O of DDS is found to be 120°, which suggests the trigonal planar geometry and sp2 hybridization state for the corresponding central S atom. The negative region surrounding both O and O atoms, opposite the positive regions of the molecule, show the complete circuits of electric field gridlines, thus distinguishing electrophilic and nucleophilic regions. The field grid lines at the negative envelope surrounding the atoms O and O atoms end up with the positive hydrogen ions [17]. The negative potential which is in the vicinity of O and O atoms is susceptible to severe electrophilic attack in the lock and key shape of the molecular electrostatic potential of DDS. Dapsone hydroxylamine caused covalent adduct formation in normal human epidermal keratinocytes [74]. Microbial iron–sulfur (Fe/S) clusters had been suggested as general targets of myeloperoxidase-derived oxidations. These effects were not caused by the destruction of the Fe/S clusters within the succinate: ubiquinone oxidoreductase but the major respiration-inhibiting lesion(s) appeared to reside at points in the respiratory chain between the Fe/S clusters and the ubiquinone reductase site [75]. The mode of DDS–DNA complex can be understood through nucleophilic properties of DDS for ubiquitination [57]. The nucleophilic/electrophilic region of DDS interacts with the electrophilic/nucleophilic region by the electronic charge transfer. The covalent adduct formation in normal human epidermal keratinocytes and the formation of hydrogen bonding in the DDS–DNA complex shows that DDS plays a role in the molecular regulation of inflammasome activators. 

The drug binding to the receptor structures is reminiscent of a pharmacological interaction between a drug and its (immune) receptor and was thus termed the p–i concept. In some patients with drug hypersensitivity, such a response occurs within hours even upon the first exposure to the drug [76]. The dapsone hypersensitivity syndrome is generally described as a combination of at least two of the following four symptoms: (i) fever, (ii) lymphadenopathy, (iii) generalized rash, and (iv) hepatitis occurring after DDS intake [22]. Its occurrence rate is subject to controversial assumptions, with estimates ranging from 2% to 12% [77]. DDS can trigger an immune response. However, metabolic activation of DDS to compete covalently with least three proteins that activate the NLRP3 inflammasome, envelope (E), ORF3a, and ORF8b, has been implicated in idiosyncratic reactions. DDS are able to decrease NF-kB signaling which to drive the transcription of inflammatory cytokines and chemokines, including IL-1b, IL-18, and IL-8, and prime NLRP3 expression to its functional level. Such DDS entities are usually characterized by the presence of ‘structural alerts’ or toxicophores [78], which can be used as substances that control the activation of the inflammasome.

### 4.3. Myeloperoxidase and Red Blood Cells’ Deformity

The peroxidase activity of heme-bound amyloid β peptides (Aβ) is associated with AD [79]. Aβ played a key role by oxidative impairing the capacity of red blood cells (RBCs) to deliver oxygen to the brain. RBC deformability leads to abnormalities in blood microcirculation [80]. Native MPO, released into the extracellular space as a result of neutrophil degranulation, is a homodimer, consisting of two identical protomers connected by a single disulfide bond, each containing light, heavy chains and heme [81]. Two MPO isoforms have distinct effects on biophysical properties of RBCs. Hemi-MPO, as well as the dimeric form, bind to the glycophorins A/B and band three protein on RBCs’ plasma membrane, which lead to reduced cell resistance to osmotic and acidic hemolysis, a reduction in cell elasticity, significant changes in cell volume, morphology, and the conductance of RBC plasma membrane ion channels. MPO, an oxidant-producing enzyme, was shown to cause RBC deformability, leading to abnormalities in the blood microcirculation [82]. Abnormalities in blood microcirculation in inflammatory foci can be induced by hemi-MPO and homodimers. This seems to reduce abnormalities in the RBC response as a regulatory mechanism that regulates the development of hemi-MPO in inflammatory foci. DDS inhibits myeloperoxidase, a mechanism that protects neurons [37].

### 4.4. Peroxymonocarbonate and Neurotoxicity

The methionine (Met) residue at position 35 in the Aβ C-terminal domain is critical for neurotoxicity, aggregation, and free radical formation initiated by the peptide [83,84,85]. Bicarbonate promotes two-electron oxidation, as mediated by hydrogen peroxide after the generation of peroxymonocarbonate (HCO_4_^−^). The bicarbonate/carbon dioxide pair stimulates one-electron oxidation mediated by the carbonate radical anion (CO_3_^●−^), which efficiently oxidizes the thioether sulfur of the Met residue to sulfoxide. DDS blocks it, so bicarbonate cannot promote two-electron oxidations mediated by hydrogen peroxide after the generation of peroxymonocarbonate (HCO_4_^−^). In addition, the bicarbonate/carbon dioxide pair cannot stimulate one-electron oxidation mediated by a carbonate radical anion (CO_3_^●−^), which efficiently oxidizes the thioether sulfur of the Met residue to sulfoxide. CO_3_^●−^ causes the one-electron oxidation of methionine residue to sulfur radical cation (MetS^●+^) [86]. Though COVID-19 patients exhibit neurological signs and symptoms, the histopathological examination of brain specimens only shows hypoxic changes, and not encephalitis or other specific brain changes that can be related to the virus. Pathogen-associated molecular patterns (PAMP) or danger-associated molecular patterns (DAMP) induced by SARS-CoV-2 may affect neurological symptoms.

### 4.5. DDS Performs Various Anti-Inflammatory Reactions

The level of parkin was decreased in the cerebellum, brain stem, substantia nigra, and striatum of aged mice. However, DDS restored the level of parkin, prevented age-dependent DA neuronal loss, and protected SH-SY5Y cells from 1-methyl-4-phenylpyridinium and hydrogen peroxide. DDS transcriptionally activated parkin via protein kinase RNA-like endoplasmic reticulum kinase-activating transcription factor 4 (ATF4) [87]. Lipopolysaccharide (LPS) distinctly activated bone marrow (BM, harvested from the femurs and tibias of C57BL/6 mice), with several distinct characteristics including high cellular activity, granulocyte changes, and a tumor necrosis factor alpha (TNF-α) production increase. Interestingly, DDS modulated the inflammatory cells, including granulocytes in LPS-treated BMs, by inducing cell death. DDS decreased the mitochondrial membrane potential of LPS-treated BMs [88] and reduced acetic acid-induced inflammatory response in rat colon tissue through the inhibition of the NF-kB signaling pathway [89]. DDS has an effect on mRNA expression and the production of cytokines in lipopolysaccharide-stimulated peripheral blood mononuclear cells. DDS suppressed mRNA expression of tumor necrosis factor (TNF)-α and significantly decreased the level of TNF-α in culture supernatant [90].

The administration of DDS with doxorubicin significantly reversed the alterations induced by doxorubicin in serum levels of CK-MB (creatine kinase myocardial band), electrocardiographic (ECG) parameters (QRS complexes: the combination of three of the graphical deflections seen on a typical ECG, RR: the time between QRS complexes and QT intervals: is defined from the beginning of the QRS complex to the end of the T wave.), papillary muscle contractility, and excitation, and the measurement of malondialdehyde (MDA), superoxide dismutase (SOD, an antioxidant enzyme), and TNF-α levels in tissue indicated that DDS significantly reduced oxidative stress and inflammation, consistent with histopathological analysis [91]. DDS has anti-inflammatory effects on LPS-mediated inflammation via the modulation of the number and function of inflammatory cells. DDS decreases MPO activity and lipid peroxidation, improves neurological function, increases the amount of spared tissue, and exerts an anti-apoptotic effect. 

Bacterial inflammagens, through gingipain protease groups and the lipopolysaccharides (LPS) of Porphyromonas gingivalis and Treponema, etc., can fuel inflammatory processes both systemically and under neuroinflammatory conditions [92,93,94,95,96]. DDS displayed myeloperoxidase inhibitor activity in saliva from subjects with periodontal disease in the sandwich test disk [97]. DDS was an anti-inflammatory agent in periodontal disease.

DDS modulates the production of inflammation-related cytokines in the spleen cells of mice, which are major immune cells, and lipopolysaccharide (LPS). Dapsone induced a proportional change in splenocyte subsets and the apoptosis of spleen cells. DDS decreased nitric oxide production in LPS-treated spleen cells in the experiment of DDS-induced production of nitric oxide (NO) and the expression of activation markers of spleen cells [98].

Familial Mediterranean Fever (FMF) caused by homozygous or compound heterozygous gain-of-function mutations in the Mediterranean fever (MEFV) gene, which encodes pyrin, an inflammasome protein [99], is an hereditary autoinflammatory disease that presents with recurrent febrile attacks and poly serositis. Pyrin—the protein involved in FMF—has a role in activating the proinflammatory cytokine interleukin (IL)-1β and colchicine is the only known treatment in this disease but nearly 5–10% of patients are resistant to colchicine [100]. In five of ten patients who prescribed with DDS, FMF attacks did not occur during at least six months (mean, 8 months and 6 days) with colchicine efficacy of 80% and 50% response. DDS may be useful as an alternative therapy in some, especially colchicine resistant patients [101]. 

It is presumed that DDS provokes an anti-inflammatory reaction in various diseases by the molecular regulation of NLRP3 inflammasome activators. This means that RBC splitting, followed by hematocrit and microfibrin formation by innate immune cells, results in various cascade reactions such as canonical‒noncanonical ubiquitylation, NLRP3 inflammasome formation, Higgins’ cascade, and iron-rich strongly magnetic nanoparticles. Perhaps Alzheimer’s disease, prion diseases, 2-DM, and Parkinson’s disease are caused by similar inflammatory reactions. 

Whether DDS has a direct effect on reducing viral pneumonia was not known before clinical studies. However, until the development of a SARS-COV-2 vaccine, DDS as a preventive drug presents a good possibility for controlling inflammation by PAMP or DAMP.

### 4.6. Korean TLR2Arg^677^Trp Variation

The mutation (TLR2Arg^677^Trp), representing the substitution of Arg for Trp at amino acid residue 677, occurs in one of the conserved regions of TLR. The major histocompatibility complex antigen loci (HLA) are the prototypical candidates for genetic susceptibility to infectious diseases [102,103]. Haplotype HLA loci variability results from selective pressure during co-evolution with pathogens. Immunologists have found that different HLA haplotypes are associated with distinct disease susceptibilities.

The repertoire of HLA molecules comprising a particular haplotype determines survival during evolution. The susceptibility to various infectious diseases, such as tuberculosis, leprosy (also known as Hansen’s disease), human immunodeficiency virus (HIV), hepatitis B, and influenza, is associated with specific HLA haplotypes [104].

However, the impairment of TLRs due to polymorphisms of TLR genes can alter the immune response to a wide variety of microbial ligands, including viruses, and polymorphisms in TLR2 and TLR4 have been linked to infectious diseases in humans. The activation of NF-κB by human TLR2Arg^677^Trp was abolished in response to Mycobacterium leprae and Mycobacterium tuberculosis [105]. TLR2 and TLR4 expression and their ligands, signaling, and functional activation are increased in diagnosed type 2 diabetes and contribute to the proinflammatory state [106]. 

TLR signaling is involved in the pathogenesis of chronic and/or idiopathic inflammatory disorders. NLR signaling results in the formation of an inflammasome and orchestrates with TLRs to induce IL-1β and IL-18, both of which are important mediators in the majority of inflammatory disorders [107]. It can be understood by using dextran sulphate sodium to activate/deactivate TLR/NLR signaling to test pro-inflammatory cytokines and immune cells and causing intestinal injuries according to microbiota [108]. Polymorphisms of TLRs and NLRs genes and markers of lipid and glucose metabolism lead to translocation to NFκB and the transcription of pro-inflammatory cytokines including IL-1β and IL-18, culminating in an inflammatory response [109]. 

Mycobacterium leprae, which induces leprosy, was assumed to be the cause of the low incidence of Alzheimer’s disease in patients with Hansen’s disease [110]. However, Hansen’s disease is successfully treated with a combination of antibiotics. Mycobacterium leprae was inactivated. These include DDS plus rifampicin, with clofazimine being added for some types of the disease. Korean patients with LL were treated using a combination of DDS with rifampicin, and clofazimine was included in some cases. Hence, the mutation in the intracellular domain of TLR2 has a role in determining the susceptibility to LL, though LL could be successfully treated using a combination of DDS with rifampicin, and clofazimine. All or part of the three antibiotics should contain an anti-inflammatory agent that works regardless of the mutation in the intracellular domain of TLR2. When only DDS was administered in the Seoul study, it was effective in treating the inflammation of dementia syndrome. 

SARS-CoV-2 can trigger TLRs. As ssRNA viruses, SARS-CoV and SARS-CoV-2 invade the cells using endosomal pathway and releases genomic RNA into the endosome [45] to bind TLR7/8 and trigger inflammatory responses in the lungs [111]. Angiotensin-converting enzyme 2 (ACE2) also contributes to inflammation in the lungs [112].

In addition to the polymorphisms in the innate immune response, MHC (major histocompatibility complex) class II polymorphism may also play a role. The processing of SARS-CoV-2 in the lysosome could make viral proteins available to MHC class II Ag presentation. This presentation could be influenced by highly polymorphic HLA-DP (a protein/peptide-antigen receptor and graft-versus-host disease antigen that is composed of 2 subunit), -DQ (a cell surface receptor protein found on antigen-presenting cells), -DR (an MHC class II cell surface receptor encoded by the human leukocyte antigen complex on chromosome 6 region 6p21.31.), and –DM (an intracellular protein involved in the mechanism of antigen presentation on antigen presenting cells (APCs) of the immune system) in modulating immune responses, as reported in other inflammatory diseases [111,113].

### 4.7. COVID-19 Is a Generalized Systemic Immune Disease

The laboratory tests conducted in the Korea National Medical Center for isolation and treatment showed mild lymphopenia in 1267 cells/mm^3^ (18.1% among white blood cells) and C-reactive protein (CRP) was 8.93 mg/dL (<0.5). In high resolution computed tomography (HRCT), bilateral patch consolidation of both lower lobes was found, showing basal lung predominance and multifocal, subpleural ground-glass opacities with interlobular septal thickening in both lungs, mainly in the right upper and middle lobes. After that, similar patterns were repeated [114].

High levels of IL-6, IL-8, and high levels of these cytokines also correlate with mortality. The activation of both the immune system and the coagulation system are not simply associated in time but, upon injury by a microorganism, immune cells are recruited and many pro-inflammatory cytokines are secreted, and these cytokines are key mediators of activation of coagulation [115]. The presence of venous thromboembolisms is observed in the lungs with severe pneumonia in some COVID-19 patients’ autopsies [116].

Moreover, SARS-CoV-2 may cause pleiotropic alterations of glucose metabolism that could complicate the pathophysiology of preexisting diabetes or lead to new mechanisms of disease [117,118]. A critical literature review suggests that the severity of SARS-CoV-2 infection is associated with the dysregulation of inflammatory immune responses, which in turn inhibits the development of protective immunity to the infection [111]. Now, COVID-19 is a multisystem inflammatory syndrome in U.S. children and adolescents [119].

SARS-CoV-2 could trigger an extreme cascade inflammatory state, which would impair the ability of the pancreas to detect glucose and balance the insulin level, and weaken the ability of the liver and muscles to sense the hormone. The NLRP3 inflammasome is implicated in a variety of human diseases including Alzheimer’s disease (AD), prion diseases, type 2 diabetes, and numerous infectious diseases. SARS-CoV-2 acts like the NLRP3 inflammasome.

### 4.8. Other Anti-Inflammatory Reactants

Dexamethasone is associated with reduced mortality risk among patients with severe COVID-19, making it the first drug to show such an effect, according to a statement by trial investigators at the University of Oxford. Over 6000 hospitalized patients were randomized to receive either dexamethasone (6 mg daily) or usual care for 10 days [120,121]. Our data reveal an incidence of hospitalization among patients with immune-mediated inflammatory disease that was consistent with that among patients with COVID-19 in the general population in New York City. Anticytokines and other immunosuppressive therapies are urgently needed to guide clinicians in the care of patients with psoriasis, rheumatoid arthritis, psoriatic arthritis, inflammatory bowel disease, and related conditions [122]. Physicians and healthcare providers should continue to use supportive measures, but also consider screening by measuring inflammatory markers, risk-categorizing individuals, treating the immune response to this virus, and studying anti-inflammatory therapies [123].

### 4.9. The Evidence of Viral Virulence Inhibition by DDS

According to studies of AIDS (Acquired Immune Deficiency Syndrome) in the 1990s, the inadvertent simultaneous administration of low doses of oral iron with DDS for the prophylaxis of Pneumocystis Carinii pneumonia in HIV-positive patients may have been associated with excess mortality [124]. A direct effect of DDS on HIV-1 replication in primary cultures of lymphocytes and monocyte-derived macrophages could explain the increased mortality rate of patients receiving DDS in addition to other factors such as the advanced stage of disease or contained iron protoxalate [125]. Lower survival in AIDS patients receiving DDS was compared with aerosolized pentamidine for secondary prophylaxis of Pneumocystis Carinii Pneumonia (PCP) [126,127]. However, DDS was effective in some patients with HIV-related thrombocytopenia [128]. DDS/pyrimethamine was as effective as aerosolized pentamidine as prophylaxis for PCP and significantly reduced the incidence of toxoplasmic encephalitis among those participants who tolerated it [129]. Iron plays a central role in oxidative stress by virtue of the generation of hydroxyl radicals via the iron-catalyzed Haber–Weiss (superoxide-driven Fenton) reaction. Oxidative stress, which, in the laboratory, can be conveniently induced by exposure to hydrogen peroxide (H_2_O_2_), induces the nuclear appearance of nuclear transcription factor NF-KB and leads to HIV-l proviral DNA transcription [130]. Viruses hijack cells in order to replicate, and efficient replication needs an iron-replete host. Some viruses selectively infect iron-acquiring cells by binding to a transferrin receptor during cell entry. Other viruses alter the expression of proteins involved in iron homeostasis, such as HFE (Homeostatic Iron Regulator) and hepcidin. In HIV-1 and hepatitis C virus infections, iron overload is associated with poor prognosis and could be partly caused by the viruses themselves [131]. Derangement in iron metabolism, in addition to oxidative stress, might have contributed to the depletion of CD4+T cell population in our subjects and this may result in the poor prognosis of the disease [132]. 

DDS is an inflammasome competitor. No one has suggested that it interferes with viral replication thus far. When viral virulence goes beyond mild symptoms, DDS may prevent it from acting as an inflammasome activator. In addition, in the case of elderly and diabetic patients, where the virus is more easily bound to cell receptors, the change in pH due to inflammation may be reduced, thereby preventing the virus from penetrating the cells. Until a vaccine is released, DDS may be a medicine that helps citizens to survive without fatal symptoms.

## 5. Conclusions

The use of therapeutics that modulate inflammation without compromising the adaptive immune response could be the most effective therapeutic strategy. As an example of this type therapeutic, DDS has multiple potent activities. The DDS–DNA complex can be used as an oral vaccine. Therefore, DDS, as an inflammasome competitor, may be effective against COVID-19. It could become one of the simplest ways to treat or prevent infectious respiratory diseases.

## Figures and Tables

**Table 1:** The clinical manifestations of SARS-CoV-2 and DDS in humans.

Clinical Manifestations	Sars-Cov-2 Symptoms	DDS Symptoms
skin vascular symptom slymphadenopathy fever	(i) violaceous macules with porcelain appearance, (ii) livedo of the trunk with chest pain and cough, (iii) violaceous macule and Raynaud’s phenomenon 10 days after fever and cough, (iv) necrotic purpura in a patient treated with leflunomide and systemic steroids for rheumatoid arthritis, (v) chilblain appearance and Raynaud’s phenomenon in a patient with anosmia, fever and cough, (vi) eruptive cherry angioma 21 days after COVID-19 healing of clinical symptoms [16]	“glandular fever”: (i) fever, (ii) lymphadenopathy, (iii) generalized rash, and (iv) hepatitis occurring after dapsone intake [22] rash, exanthema/erythema, erythroderma, mucosal involvement [14] papular or exfoliative dermatitis, generalized lymphadenopathy [23]
hypersensitivity reactions	SARS-CoV-2 symptoms are like a severe idiosyncratic DDS reaction characterized by the clinical triad of fever, rash, and systemic involvement, which can cause severe organ (heart, kidney, lung, brain, etc.) dysfunction [24]	the syndrome is a severe idiosyncratic DDS reaction characterized by the clinical triad of fever, rash, and systemic involvement (most commonly of the liver and the hematologic system), which can cause severe organ dysfunction [13]
hematology laboratory	focal fibrin clusters mixed with mononuclear inflammatory cells, decreased eosinophils, decreased lymphocytes, increased neutrophils [25] lymphopenia, leukocytosis, neutrophilia, thrombocytopenia [26]	leukocytosis, eosinophilia [14], resembling a mononucleosis infection [23]
anemia	thrombocytopenia, consumptive coagulopathy [26]	hemolytic anemia and methemoglobinemia [12]
liver disease, pancreatic disease	clinically significant liver injury is uncommon [27], pancreatic cells highly express ACE2 [28]	hepatitis/liver toxicity [23], cholangitis, colitis, thyroiditis [29], pancreatitis and pleural effusion [30]
renal disease	severe collapsing focal segmental glomerulosclerosis, acute tubular necrosis [31]	acute renal failure [23],
cardiac disease	acute myocardial injury and chronic damage to the cardiovascular system [32]	myocarditis, dapsone-induced hypersensitivity syndrome-associated complete atrioventricular block [29], myocardial injury [33]
pulmonary disease	coronavirus disease (COVID-19)-related pneumonia [24,32]	pneumonitis [29], pneumonia or multiple organ failure [33]
neurologic disease	large-vessel stroke [34] encephalopathy, prominent agitation and confusion, and corticospinal tract signs [35,36]	recovery of dementia syndrome following treatment of brain inflammation [37]

**Table 2:** Hansen’s disease recurrences and prevalence of respiratory infectious disease.

	Cumulative Recurrence Risk Index				Infectious Respiratory Diseases	
Year	Inactive Patient	Number of Recurrent Patients	Annual Recurrence Rate (%)	DDS Intake (100 mg)	Infected Person	Remarks (Report)
2002 SARS-CoV	16,712	10	0.05		-(0 person)	(no prevalence)
2003	16,283	6	0.03		-	-
2004	15,797	6	0.03		-	-
2005	15,350	5	0.03		-	-
2006	14,851	5	0.03		-	-
2007	14,321	3	0.02		-	-
2008	13,875	3	0.02		-	-
2009 Influenza A virus subtype H1N1	13,422	3	0.02		-(0 person)	(no prevalence)
2010	13,033	7	0.05		-	-
2011	12,582	3	0.02		-	-
2012	12,068	1	0.01		-	-
2013	11,595	1	0.01		-	-
2014	11,104	1	0.01		-	-
2015 MERS	10,653	1	0.01		-(0 person)	(no prevalence)
2016	10,236	-	-		-	-
2017	9908	-	-		-	-
2018	9503	1	0.01	2019–2020.6. 3814(person)/9134(total)	-	-
2019 SARS-CoV-2	9160	2	0.02		-(0 person)	(no prevalence)
–2020. 6.30. SARS-CoV-2					-(0 person)	(no prevalence)

**Table 3:** Age distribution of Hansen’s disease in Korea (2019): Hansen’s disease status and major indicators.

	Total	Age					Average Age				
		0–19	20–39	40–59	60–79	80–	SUM	Treatment Place			
								Home	Town	Nursing	Hospital
total	9288 (100%)	-	18 (0.2%)	545 (5.9%)	5147 (55.4%)	3578 (38.5%)	79	78	80	81	78
Active leprosy patients	128 (100%)	-	7 (5.5%)	9 (7.0%)	79 (61.7%)	33 (25.8%)	71	73	68	65	77

**Table 4:** Treatment of neuroinflammation with anti-Alzheimer’s disease drug (AAD) used as a new biomarker of numeric clinical staging (NCS)—a new biomarker of numeric clinical staging (NCS) is denoted as (D). (D) (added NCS is italicized) (Supplement. 2).

Year	NCS	Supplementary Material
2007	Stage 2	Supplement_6.pdf (page 2) of online suppl. 6 [21] (suppl.2)
2008.02.05	Stage 3	Supplement_4.pdf (page 1) of online suppl. 4 [21] (suppl.2)
*Seoul study—DDS*		
*DDS supply was suspended in Korea from 2016*		
2018.06.27	Stage 4	Supplement_6.pdf (pages 1–2) of online suppl. 6 [21]
*2018.06.27–10.01*	*Stage 6*	*D side effects Dr prescribed (D) (suppl.3)*
*2018.10.–*	*Stage 5*	*Stop AAD*
2018.11.06	Stage 5	Supplement_1.pdf (pages 1–2) of online suppl. 1 [21] (suppl.2)
*2018.11.06–11.16 11.21*	*Stage 6*	*D’s side effects (suppl.3)*, Supplement_2.pdf (page 1) of online suppl. 2 [21] (suppl.2)
*2018.11.22–*	*Stage 5*	*Stop AAD*
*The Korea Orphan and Essential Drug Center imported and supplied DDS*		
*2018.11.28*		*DDS*, Supplement_3.pdf (page 1) of online suppl. 3 [21] (suppl.2)
2019.01.14	Stage 3	Supplement_1.pdf (pages 3–5) of online suppl. 1 [21] (suppl.2)
2020.03.18	Stage 3	Seoul cohort—Present, DDS supply began again in Korea from January 2020. Seoul cohort prescribed DDS from 150 mg to 300 mg as an inflammasome competitor

## Supplementary Materials

Supplementary materials can be found at https://www.mdpi.com/1422-0067/21/17/5953/s1, 1. Volunteer Certificate; 2. J., Lee; S., Choi; C.J., Lee; S., Oh (2020): Supplementary Material for: Recovery of Dementia Syndrome following Treatment of Brain Inflammation. https://doi.org/10.6084/m9.figshare.11671407.v1 [21]. Embedded <iframe src=“https://widgets.figshare.com/articles/11671407/embed?show_title=1” width=“568” height=“351” allowfullscreen=“true” frameborder=“0”></iframe>; 3. D’s side effects.

## Abbreviations

AD	Alzheimer’s disease
DDS	4,4′-Diaminodiphenyl sulfone (dapsone)
LL	Lepromatous leprosy
MADDS	Monoacetyldapsone
MCI	Mild cognitive impairment
PD	Parkinson’s disease
NLRP3	NOD-, LRR- and pyrin domains-containing protein 3
MPO	Myeloperoxidase
TLR	Toll-like receptor

## References

[1] Mahase E. (2020). Coronavirus: Covid-19 has killed more people than SARS and MERS combined, despite lower case fatality rate. BMJ.

[2] Graham R.L., Baric R.S. (2010). Recombination, reservoirs, and the modular spike: Mechanisms of coronavirus cross-species transmission. J. Virol..

[3] Raj V.S., Mou H., Smits S.L., Dekkers D.H., Müller M.A., Dijkman R., Muth D., Demmers J.A., Zaki A., Fouchier R.A. (2013). Dipeptidyl peptidase 4 is a functional receptor for the emerging human coronavirus-EMC. Nature.

[4] Yuan F.F., Boehm I., Chan P.K.S., Marks K., Tang J.W., Hui D.S.C., Sung J.J.Y., Dyer W.B., Geczy A.F., Sullivan J.S. (2007). High Prevalence of the CD14-159CC Genotype in Patients Infected with Severe Acute Respiratory Syndrome-Associated Coronavirus. Clin. Vaccine Immunol..

[5] Conti P., Ronconi G., Caraffa A., Gallenga C., Ross R., Frydas I., Kritas S. (2020). Induction of pro-inflammatory cytokines (IL-1 and IL-6) and lung inflammation by COVID-19: Anti-inflammatory strategies. J. Biol. Regul. Homeost Agents.

[6] Ichinohe T., Yamazaki T., Koshiba T., Yanagi Y. (2013). Mitochondrial protein mitofusin 2 is required for NLRP3 inflammasome activation after RNA virus infection. Proc. Natl. Acad. Sci. USA.

[7] Park S., Juliana C., Hong S., Datta P., Hwang I., Fernandes-Alnemri T., Yu J.-W., Alnemri E.S. (2013). The mitochondrial antiviral protein MAVS associates with NLRP3 and regulates its inflammasome activity. J. Immunol..

[8] Friker L.L., Scheiblich H., Hochheiser I.V., Brinkschulte R., Riedel D., Latz E., Geyer M., Heneka M.T. (2020). β-Amyloid Clustering around ASC Fibrils Boosts Its Toxicity in Microglia. Cell Rep..

[9] Swanson K.V., Deng M., Ting J.P.-Y. (2019). The NLRP3 inflammasome: Molecular activation and regulation to therapeutics. Nat. Rev. Immunol..

[10] Lowe J. (1950). Treatment of leprosy with diamino-diphenyl sulphone by mouth. Lancet.

[11] Zhu Y.I., Stiller M.J. (2001). Dapsone and sulfones in dermatology: Overview and update. J. Am. Acad. Dermatol..

[12] Wozel V.G. (2010). Innovative use of dapsone. Dermatol. Clin..

[13] Zhang F.R., Liu H., Irwanto A., Fu X.A., Li Y., Yu G.Q., Yu Y.X., Chen M.F., Low H.Q., Li J.H. (2013). HLA-B*13:01 and the Dapsone Hypersensitivity Syndrome. N. Engl. J. Med..

[14] Schmitt J., Lorenz M., Wozel G. (2012). Hypersensitivity Reactions to Dapsone: A Systematic Review. Acta Derm. Venereol..

[15] Kim J.-M., Chung Y.-S., Jo H.J., Lee N.-J., Kim M.S., Woo S.H., Park S., Kim J.W., Kim H.M., Han M.-G. (2020). Identification of Coronavirus Isolated from a Patient in Korea with COVID-19. Osong Public Health Res. Perspect..

[16] Bouaziz J.D., Duong T.A., Jachiet M., Velter C., Lestang P., Cassius C., Arsouze A., Domergue Than Trong E., Bagot M., Begon E. (2020). Vascular skin symptoms in COVID-19: A French observational study. J. Eur. Acad. Dermatol. Venereol..

[17] Rajendran N.D., Natarajan Mookan I.S., Mookan S.B., Munusamy G., Gurudeeban S., Kaliamurthi S. (2020). A theoretical study of chemical bonding and topological and electrostatic properties of the anti-leprosy drug dapsone. J. Mol. Modeling.

[18] Parr R.G. (1980). Density functional theory of atoms and molecules. Horizons of Quantum Chemistry.

[19] Cho Y., Shim E., Lee K.-S., Park S.C. (2014). Mortality profiles of leprosy-affected elderly in Korea: A demographic perspective. Asia Pac. E J. Health Soc. Sci..

[20] Ahn Y.-H., Park H., Kweon S.-S. (2020). Causes of Death among Persons Affected by Leprosy in Korea, 2010–2013. Am. J. Trop. Med. Hyg..

[21] Lee J., Choi S., Lee C.J., Oh S. (2020). Supplementary Material for: Recovery of Dementia Syndrome following Treatment of Brain Inflammation. Dement. Geriatr. Cogn. Disord. EXTRA.

[22] JH R., Smith T. (1989). Increased incidence in leprosy of hypersensitivity reactions to dapsone after introduction of multidrug therapy. Lepr. Rev..

[23] Alves-Rodrigues E.N., Ribeiro L.C., Silva M.D., Takiuchi A., Fontes C.J.F. (2005). Dapsone syndrome with acute renal failure during leprosy treatment: Case report. Braz. J. Infect. Dis..

[24] Singhal T. (2020). A Review of Coronavirus Disease-2019 (COVID-19). Indian J. Pediatr..

[25] Tian S., Hu W., Niu L., Liu H., Xu H., Xiao S.-Y. (2020). Pulmonary pathology of early phase 2019 novel coronavirus (COVID-19) pneumonia in two patients with lung cancer. J. Thorac. Oncol..

[26] Frater J.L., Zini G., D’Onofrio G., Rogers H.J. (2020). COVID-19 and the clinical hematology laboratory. Int. J. Lab. Hematol..

[27] Bangash M.N., Patel J., Parekh D. (2020). COVID-19 and the liver: Little cause for concern. Lancet Gastroenterol. Hepatol..

[28] Liu F., Long X., Zou W., Fang M., Wu W., Li W., Zhang B., Zhang W., Chen X., Zhang Z. (2020). Highly ACE2 Expression in Pancreas May Cause Pancreas Damage after SARS-CoV-2 Infection. medRxiv.

[29] Zhu K., He F., Jin N., Lou J., Cheng H. (2009). Complete atrioventricular block associated with dapsone therapy: A rare complication of dapsone-induced hypersensitivity syndrome. J. Clin. Pharm. Ther..

[30] Ghishan F.K. (1998). The sulfone syndrome complicated by pancreatitis and pleural effusion in an adolescent receiving dapsone for treatment of acne vulgaris. J. Pediatr. Gastroenterol. Nutr..

[31] Kissling S., Rotman S., Gerber C., Halfon M., Lamoth F., Comte D., Lhopitallier L., Sadallah S., Fakhouri F. (2020). Collapsing glomerulopathy in a COVID-19 patient. Kidney Int..

[32] Zheng Y.-Y., Ma Y.-T., Zhang J.-Y., Xie X. (2020). COVID-19 and the cardiovascular system. Nat. Rev. Cardiol..

[33] Kang K.S., Kim H.I., Kim O.H., Cha K.C., Kim H., Lee K.H., Hwang S.O., Cha Y.S. (2016). Clinical outcomes of adverse cardiovascular events in patients with acute dapsone poisoning. Clin. Exp. Emerg. Med..

[34] Oxley T.J., Mocco J., Majidi S., Kellner C.P., Shoirah H., Singh I.P., De Leacy R.A., Shigematsu T., Ladner T.R., Yaeger K.A. (2020). Large-Vessel Stroke as a Presenting Feature of Covid-19 in the Young. N. Engl. J. Med..

[35] Helms J., Kremer S., Merdji H., Clere-Jehl R., Schenck M., Kummerlen C., Collange O., Boulay C., Fafi-Kremer S., Ohana M. (2020). Neurologic Features in Severe SARS-CoV-2 Infection. N. Engl. J. Med..

[36] Kast R.E. (2020). Dapsone as treatment adjunct in ARDS. Exp. Lung Res..

[37] Lee J.-H., Choi S.-H., Lee C.J., Oh S.-S. (2020). Recovery of Dementia Syndrome following Treatment of Brain Inflammation. Dement. Geriatr. Cogn. Disord. Extra.

[38] Moura S.L., Fernandes G.F.S., Machado F.B.C., Ferrão L.F.A. (2020). Theoretical and experimental electronic spectra of neutral, monoprotonated and diprotonated dapsone. Theor. Chem. Acc..

[39] Mendes A.P., Schalcher T.R., Barros T.G., Almeida E.D., Maia C.S., Barros C.A., Monteiro M.C., Borges R.S. (2011). A Geometric and Electronic Study of Dapsone. J. Comput. Theor. Nanosci..

[40] McDowell G.S., Philpott A. (2013). Non-canonical ubiquitylation: Mechanisms and consequences. Int. J. Biochem. Cell. Biol..

[41] McClellan A.J., Laugesen S.H., Ellgaard L. (2019). Cellular functions and molecular mechanisms of non-lysine ubiquitination. Open Biol..

[42] Hyne J.B., Greidanus J.W. (1969). Nuclear magnetic resonance study of intramolecular electronic effects in diphenyl sulfides, sulfoxides, and sulfones. Can. J. Chem..

[43] Foley J.F. (2016). Serine ubiquitylation. Sci. Signal..

[44] Kume K., Iizumi Y., Shimada M., Ito Y., Kishi T., Yamaguchi Y., Handa H. (2010). Role of N-end rule ubiquitin ligases UBR1 and UBR2 in regulating the leucine-mTOR signaling pathway. Genes Cells.

[45] Simmons G., Reeves J.D., Rennekamp A.J., Amberg S.M., Piefer A.J., Bates P. (2004). Characterization of severe acute respiratory syndrome-associated coronavirus (SARS-CoV) spike glycoprotein-mediated viral entry. Proc. Natl. Acad. Sci. USA.

[46] Wang M., Cao R., Zhang L., Yang X., Liu J., Xu M., Shi Z., Hu Z., Xiao G. (2020). Remdesivir and chloroquine effectively inhibit the recently emerged novel coronavirus (2019-nCoV) in vitro. Cell Res..

[47] Ducatelle R., Hoorens J. (1984). Significance of lysosomes in the morphogenesis of coronaviruses. Arch. Virol..

[48] Bozeman P.M., Learn D.B., Thomas E.L. (1992). Inhibition of the human leukocyte enzymes myeloperoxidase and eosinophil peroxidase by dapsone. Biochem. Pharmacol..

[49] Van Zyl J.M., Basson K., Kriegler A., van der Walt B.J. (1991). Mechanisms by which clofazimine and dapsone inhibit the myeloperoxidase system: A possible correlation with their anti-inflammatory properties. Biochem. Pharmacol..

[50] Uetrecht J.P., Shear N.H., Zahid N. (1993). N-chlorination of sulfamethoxazole and dapsone by the myeloperoxidase system. Drug Metab. Dispos..

[51] Kim S.-K., Lee S.-B., Kang T.-J., Chae G.-T. (2003). Detection of gene mutations related with drug resistance inMycobacterium lepraefrom leprosy patients using Touch-Down (TD) PCR. FEMS Immunol. Med. Microbiol..

[52] Kang T.-J., Chae G.-T. (2001). Detection of Toll-like receptor 2 (TLR2) mutation in the lepromatous leprosy patients. FEMS Immunol. Med. Microbiol..

[53] Yap J.K.Y., Moriyama M., Iwasaki A. (2020). Inflammasomes and Pyroptosis as Therapeutic Targets for COVID-19. J. Immunol..

[54] Shi C.-S., Nabar N.R., Huang N.-N., Kehrl J.H. (2019). SARS-Coronavirus Open Reading Frame-8b triggers intracellular stress pathways and activates NLRP3 inflammasomes. Cell Death Discov..

[55] Chan J.F.-W., Kok K.-H., Zhu Z., Chu H., To K.K.-W., Yuan S., Yuen K.-Y. (2020). Genomic characterization of the 2019 novel human-pathogenic coronavirus isolated from a patient with atypical pneumonia after visiting Wuhan. Emerg. Microbes Infect..

[56] Lu R., Zhao X., Li J., Niu P., Yang B., Wu H., Wang W., Song H., Huang B., Zhu N. (2020). Genomic characterisation and epidemiology of 2019 novel coronavirus: Implications for virus origins and receptor binding. Lancet.

[57] Chakraborty A., Panda A., Ghosh R., Biswas A. (2019). DNA minor groove binding of a well known anti-mycobacterial drug dapsone: A spectroscopic, viscometric and molecular docking study. Arch. Biochem. Biophys..

[58] Lee K.H., Park J.H., Kim D.H., Hwang J., Lee G., Hyun J.S., Heo S.T., Choi J.H., Kim M., Kim M. (2017). Dapsone as a potential treatment option for Henoch-Schönlein Purpura (HSP). Med. Hypotheses.

[59] Ahmed F. (2010). Epigenetics: Tales of adversity. Nature.

[60] Demoinet E., Li S., Roy R. (2017). AMPK blocks starvation-inducible transgenerational defects in Caenorhabditis elegans. Proc. Natl. Acad. Sci. USA.

[61] Yamaza H., Chiba T., Higami Y., Shimokawa I. (2002). Lifespan extension by caloric restriction: An aspect of energy metabolism. Microsc. Res. Tech..

[62] Gibson M., Rogers C., Murrell D. (2020). Successful dapsone therapy in inherited Epidermolysis Bullosa. J. Eur. Acad. Dermatol. Venereol..

[63] Ghaoui N., Hanna E., Abbas O., Kibbi A.G., Kurban M. (2020). Update on the use of dapsone in dermatology. Int. J. Dermatol..

[64] Diaz-Ruiz A., Zavala C., Montes S., Ortiz-Plata A., Salgado-Ceballos H., Orozco-Suarez S., Nava-Ruiz C., Pérez-Neri I., Perez-Severiano F., Ríos C. (2008). Antioxidant, antiinflammatory and antiapoptotic effects of dapsone in a model of brain ischemia/reperfusion in rats. J. Neurosci. Res..

[65] Mahale A., Kumar R., Sarode L.P., Gakare S., Prakash A., Ugale R.R. (2020). Dapsone prolong delayed excitotoxic neuronal cell death by interacting with proapoptotic/survival signaling proteins. J. Stroke Cerebrovasc. Dis..

[66] Yang N., Li L., Li Z., Ni C., Cao Y., Liu T., Tian M., Chui D., Guo X. (2017). Protective effect of dapsone on cognitive impairment induced by propofol involves hippocampal autophagy. Neurosci. Lett..

[67] Ríos C., Orozco-Suarez S., Salgado-Ceballos H., Mendez-Armenta M., Nava-Ruiz C., Santander I., Barón-Flores V., Caram-Salas N., Diaz-Ruiz A. (2015). Anti-apoptotic effects of dapsone after spinal cord injury in rats. Neurochem. Res..

[68] Namba Y., Kawatsu K., Izumi S., Ueki A., Ikeda K. (1992). Neurofibrillary tangles and senile plaques in brain of elderly leprosy patients. Lancet.

[69] Kimura T., Goto M. (1993). Existence of senile plaques in the brains of elderly leprosy patients. Lancet.

[70] Chui D.-H., Tabira T., Izumi S., Koya G., Ogata J. (1994). Decreased beta-amyloid and increased abnormal Tau deposition in the brain of aged patients with leprosy. Am. J. Pathol..

[71] Appleby B.S., Cummings L.J. (2013). Discovering new treatments for Alzheimer’s disease by repurposing approved medications. Curr. Top. Med. Chem..

[72] Bain A. (2002). Alzheimer disease: Dapsone phase 2 trial results reported. Immune Netw. Ltd. Press Release.

[73] Imbimbo B.P., Solfrizzi V., Panza F. (2010). Are NSAIDs useful to treat Alzheimer’s disease or mild cognitive impairment?. Front. Aging Neurosci..

[74] Reilly T.P., Lash L.H., Doll M.A., Hein D.W., Woster P.M., Svensson C.K. (2000). A role for bioactivation and covalent binding within epidermal keratinocytes in sulfonamide-induced cutaneous drug reactions. J. Investig. Dermatol..

[75] Hurst J.K., Barrette W.C., Michel B.R., Rosen H. (1991). Hypochlorous acid and myeloperoxidase-catalyzed oxidation of iron-slfur clusters in bacterial respiratory dehydrogenases. Eur. J. Biochem..

[76] Posadas S., Pichler W. (2007). Delayed drug hypersensitivity reactions-new concepts. Clin. Exp. Allergy J. Br. Soc. Allergy Clin. Immunol..

[77] Smith W.C.S. (1988). Are hypersensitivity reactions to dapsone becoming more frequent?. Lepr. Rev..

[78] Stepan A.F., Walker D.P., Bauman J., Price D.A., Baillie T.A., Kalgutkar A.S., Aleo M.D. (2011). Structural alert/reactive metabolite concept as applied in medicinal chemistry to mitigate the risk of idiosyncratic drug toxicity: A perspective based on the critical examination of trends in the top 200 drugs marketed in the United States. Chem. Res. Toxicol..

[79] Roy M., Pal I., Nath A.K., Dey S.G. (2020). Peroxidase activity of heme bound amyloid β peptides associated with Alzheimer’s disease. Chem. Commun..

[80] Kiko T., Nakagawa K., Satoh A., Tsuduki T., Furukawa K., Arai H. (2012). Amyloid β Levels in Human Red Blood Cells. PLoS ONE.

[81] Blair-Johnson M., Fiedler T., Fenna R. (2001). Human myeloperoxidase: Structure of a cyanide complex and its interaction with bromide and thiocyanate substrates at 1.9 Å resolution. Biochemistry.

[82] Shamova E.V., Gorudko I.V., Grigorieva D.V., Sokolov A.V., Kokhan A.U., Melnikova G.B., Yafremau N.A., Gusev S.A., Sveshnikova A.N., Vasilyev V.B. (2020). The effect of myeloperoxidase isoforms on biophysical properties of red blood cells. Mol. Cell. Biochem..

[83] Varadarajan S., Yatin S., Kanski J., Jahanshahi F., Butterfield D.A. (1999). Methionine residue 35 is important in amyloid β-peptide-associated free radical oxidative stress. Brain Res. Bull..

[84] Vogt W. (1995). Oxidation of methionyl residues in proteins: Tools, targets, and reversal. Free Radic. Biol. Med..

[85] Enache T.A., Oliveira-Brett A.M. (2017). Alzheimer’s disease amyloid beta peptides in vitro electrochemical oxidation. Bioelectrochemistry.

[86] Francioso A., Baseggio Conrado A., Blarzino C., Foppoli C., Montanari E., Dinarelli S., Giorgi A., Mosca L., Fontana M. (2020). One-and Two-Electron Oxidations of β-Amyloid25-35 by Carbonate Radical Anion (CO3•−) and Peroxymonocarbonate (HCO4−): Role of Sulfur in Radical Reactions and Peptide Aggregation. Molecules.

[87] Lee Y.-I., Kang H., Ha Y.W., Chang K.-Y., Cho S.-C., Song S.O., Kim H., Jo A., Khang R., Choi J.-Y. (2016). Diaminodiphenyl sulfone–induced parkin ameliorates age-dependent dopaminergic neuronal loss. Neurobiol. Aging.

[88] Kwon M.-J., Joo H.-G. (2018). Dapsone modulates lipopolysaccharide-activated bone marrow cells by inducing cell death and down-regulating tumor necrosis factor-α production. J. Vet. Sci..

[89] Rashidian A., Rashki A., Abdollahi A., Haddadi N.-S., Chamanara M., Mumtaz F., Dehpour A.R. (2019). Dapsone reduced acetic acid-induced inflammatory response in rat colon tissue through inhibition of NF-kB signaling pathway. Immunopharmacol. Immunotoxicol..

[90] Abe M., Shimizu A., Yokoyama Y., Takeuchi Y., Ishikawa O. (2008). A possible inhibitory action of diaminodiphenyl sulfone on tumour necrosis factor-α production from activated mononuclear cells on cutaneous lupus erythematosus. Clin. Exp. Dermatol. Exp. Dermatol..

[91] Sheibani M., Nezamoleslami S., Faghir-Ghanesefat H., hossein Emami A., Dehpour A.R. (2020). Cardioprotective effects of dapsone against doxorubicin-induced cardiotoxicity in rats. Cancer Chemother. Pharmacol..

[92] Riviere G.R., Riviere K., Smith K. (2002). Molecular and immunological evidence of oral Treponema in the human brain and their association with Alzheimer’s disease. Oral Microbiol. Immunol..

[93] Kamer A.R., Dasanayake A.P., Craig R.G., Glodzik-Sobanska L., Bry M., De Leon M.J. (2008). Alzheimer’s disease and peripheral infections: The possible contribution from periodontal infections, model and hypothesis. J. Alzheimers Dis..

[94] Leira Y., Dominguez C., Seoane J., Seoane-Romero J., Pías-Peleteiro J.M., Takkouche B., Blanco J., Aldrey J.M. (2017). Is periodontal disease associated with Alzheimer’s disease? A systematic review with meta-analysis. Neuroepidemiology.

[95] Dominy S.S., Lynch C., Ermini F., Benedyk M., Marczyk A., Konradi A., Nguyen M., Haditsch U., Raha D., Griffin C. (2019). Porphyromonas gingivalis in Alzheimer’s disease brains: Evidence for disease causation and treatment with small-molecule inhibitors. Sci. Adv..

[96] Ebaid D., Crewther S.G. (2019). Visual information processing in young and older adults. Front. Aging Neurosci..

[97] Sakamoto W., Fujii Y., Kanehira T., Asano K., Izumi H. (2008). A novel assay system for myeloperoxidase activity in whole saliva. Clin. Biochem..

[98] Moon S.-Y., Joo H.-G. (2015). Anti-inflammatory effects of 4,4′-diaminodiphenyl sulfone (dapsone) in lipopolysaccharide-treated spleen cells: Selective inhibition of inflammation-related cytokines. Korean J. Vet. Res..

[99] Park Y.H., Remmers E.F., Lee W., Ombrello A.K., Chung L.K., Shilei Z., Stone D.L., Ivanov M.I., Loeven N.A., Barron K.S. (2020). Ancient familial Mediterranean fever mutations in human pyrin and resistance to Yersinia pestis. Nat. Immunol..

[100] Calligaris L., Marchetti F., Tommasini A., Ventura A. (2008). The efficacy of anakinra in an adolescent with colchicine-resistant familial Mediterranean fever. Eur. J. Pediatr..

[101] Salehzadeh F., Jahangiri S., Mohammadi E. (2012). Dapsone as an alternative therapy in children with familial Mediterranean fever. Iran. J. Pediatr..

[102] Blackwell J.M., Jamieson S.E., Burgner D. (2009). HLA and infectious diseases. Clin. Microbiol. Rev..

[103] Matzaraki V., Kumar V., Wijmenga C., Zhernakova A. (2017). The MHC locus and genetic susceptibility to autoimmune and infectious diseases. Genome Biol..

[104] Shi Y., Wang Y., Shao C., Huang J., Gan J., Huang X., Bucci E., Piacentini M., Ippolito G., Melino G. (2020). COVID-19 infection: The perspectives on immune responses. Cell Death Differ..

[105] Bochud P.-Y., Hawn T.R., Aderem A. (2003). Cutting Edge: A Toll-Like Receptor 2 Polymorphism That Is Associated with Lepromatous Leprosy Is Unable to Mediate Mycobacterial Signaling. J. Immunol..

[106] Dasu M.R., Devaraj S., Park S., Jialal I. (2010). Increased Toll-Like Receptor (TLR) Activation and TLR Ligands in Recently Diagnosed Type 2 Diabetic Subjects. Diabetes Care.

[107] Gomes T.A., Leite N., Tureck L., de Souza R., Titski A., Milano-Gai G., Lazarotto L., da Silva L., Furtado-Alle L. (2019). Association between Toll-like receptors (TLR) and NOD-like receptor (NLR) polymorphisms and lipid and glucose metabolism. Gene.

[108] Fukata M., Vamadevan A., Abreu M. (2009). Toll-like receptors (TLRs) and Nod-like receptors (NLRs) in inflammatory disorders. Semin. Immunol..

[109] Xiao Y., Yan H., Diao H., Yu B., He J., Yu J., Zheng P., Mao X., Luo Y., Chen D. (2017). Early Gut Microbiota Intervention Suppresses DSS-Induced Inflammatory Responses by Deactivating TLR/NLR Signalling in Pigs. Sci. Rep..

[110] Endoh M., Kunishita T., Tabira T. (1999). No effect of anti-leprosy drugs in the prevention of Alzheimer’s disease and β-amyloid neurotoxicity. J. Neurol. Sci..

[111] Manjili R.H., Zarei M., Habibi M., Manjili M.H. (2020). COVID-19 as an Acute Inflammatory Disease. J. Immunol..

[112] Hoffmann M., Kleine-Weber H., Schroeder S., Krüger N., Herrler T., Erichsen S., Schiergens T.S., Herrler G., Wu N.-H., Nitsche A. (2020). SARS-CoV-2 Cell Entry Depends on ACE2 and TMPRSS2 and Is Blocked by a Clinically Proven Protease Inhibitor. Cell.

[113] Wu X.-L., Li Z.-Y., Bi X.-Y., Zhao H., Zhao J.-J., Zhou J.-G., Han Y., Huang Z., Zhang Y.-F., Cai J.-Q. (2018). Human leukocyte antigen gene polymorphisms are associated with systemic inflammation in hepatitis B virus-related hepatocellular carcinoma. Cancer Manag. Res..

[114] Kim J.Y., Ko J.-H., Kim Y., Kim Y.-J., Kim J.-M., Chung Y.-S., Kim H.M., Han M.-G., Kim S.Y., Chin B.S. (2020). Viral Load Kinetics of SARS-CoV-2 Infection in First Two Patients in Korea. J. Korean Med. Sci..

[115] Bester J., Matshailwe C., Pretorius E. (2018). Simultaneous presence of hypercoagulation and increased clot lysis time due to IL-1β, IL-6 and IL-8. Cytokine.

[116] Wichmann D., Sperhake J.-P., Lütgehetmann M., Steurer S., Edler C., Heinemann A., Heinrich F., Mushumba H., Kniep I., Schröder A.S. (2020). Autopsy findings and venous thromboembolism in patients with COVID-19: A prospective cohort study. Ann. Intern. Med..

[117] Rubino F., Amiel S.A., Zimmet P., Alberti G., Bornstein S., Eckel R.H., Mingrone G., Boehm B., Cooper M.E., Chai Z. (2020). New-Onset Diabetes in Covid-19. N. Engl. J. Med..

[118] Yang L., Han Y., Nilsson-Payant B.E., Gupta V., Wang P., Duan X., Tang X., Zhu J., Zhao Z., Jaffré F. (2020). A Human Pluripotent Stem Cell-based Platform to Study SARS-CoV-2 Tropism and Model Virus Infection in Human Cells and Organoids. Cell Stem. Cell.

[119] Feldstein L.R., Rose E.B., Horwitz S.M., Collins J.P., Newhams M.M., Son M.B.F., Newburger J.W., Kleinman L.C., Heidemann S.M., Martin A.A. (2020). Multisystem Inflammatory Syndrome in U.S. Children and Adolescents. N. Engl. J. Med..

[120] Juillet G., Oxford University (2020). Low-Cost Dexamethasone Reduces Death by up to One Third in Hospitalised Patients with Severe Respiratory Complications of COVID-19.

[121] Shang L., Zhao J., Hu Y., Du R., Cao B. (2020). On the use of corticosteroids for 2019-nCoV pneumonia. Lancet.

[122] Haberman R., Axelrad J., Chen A., Castillo R., Yan D., Izmirly P., Neimann A., Adhikari S., Hudesman D., Scher J.U. (2020). Covid-19 in immune-mediated inflammatory diseases—Case series from New York. N. Engl. J. Med..

[123] Kivela P. (2020). Paradigm Shift for COVID-19 Response: Identifying High-risk Individuals and Treating Inflammation. West. J. Emerg. Med..

[124] Gordeuk V., Delanghe J., Langlois M., Boelaert J. (2001). Iron status and the outcome of HIV infection: An overview. J. Clin. Virol. Off. Publ. Pan Am. Soc. Clin. Virol..

[125] Duval X., Clayette P., Dereuddre-Bosquet N., Fretier P., Martin M., Salmon-Céron D., Gras G., Vildé J., Dormont D. (1997). Dapsone and HIV-1 replication in primary cultures of lymphocytes and monocyte-derived macrophages. Aids.

[126] Salmon-Ceron D., Fontbonne A., Saba J., May T., Raffi F., Chidiac C., Patey O., Aboulker J., Schwartz D., Vilde J. (1995). Lower survival in AIDS patients receiving dapsone compared with aerosolized pentamidine for secondary prophylaxis of Pneumocystis carinii pneumonia. J. Infect. Dis..

[127] Hughes W.T. (1998). Use of Dapsone in the Prevention and Treatment of Pneumocystis carinii Pneumonia: A Review. Clin. Infect. Dis..

[128] Durand J.M., Lefèvre P., Hovette P., Issifi S., Mongin M. (1991). Dapsone for thrombocytopenic purpura related to human immunodeficiency virus infection. Am. J. Med..

[129] Opravil M., Hirschel B., Lazzarin A., Heald A., Pechère M., Rüttimann S., Iten A., von Overbeck J., Oertle D., Praz G. (1995). Once-Weekly Administration of Dapsone/Pyrimethamine vs. Aerosolized Pentamidine as Combined Prophylaxis for Pneumocystis carinii Pneumonia and Toxoplasmic Encephalitis in Human Immunodeficiency Virus-Infected Patients. Clin. Infect. Dis..

[130] Boelaert J.R., Piette J., Weinberg G.A., Sappey C., Weinberg E.D., Rich E.A., Abbud R.A. (1996). Iron and Oxidative Stress as a Mechanism for the Enhanced Production of Human Immunodeficiency Virus by Alveolar Macrophages from Otherwise Healthy Cigarette Smokers [with Reply]. J. Infect. Dis..

[131] Drakesmith H., Prentice A. (2008). Viral infection and iron metabolism. Nat. Rev. Microbiol..

[132] Banjoko S.O., Oseni F.A., Togun R.A., Onayemi O., Emma-Okon B.O., Fakunle J.B. (2012). Iron status in HIV-1 infection: Implications in disease pathology. BMC Clin. Pathol..

