# Supplementary material for: 4,4′-Diaminodiphenyl Sulfone (DDS) as an Inflammasome Competitor
Source: Int J Mol Sci. 2020 Aug 19;21(17):5953. doi: 10.3390/ijms21175953 (PMC7503668; doi:10.3390/ijms21175953)
Supplement: Supplementary file 1 [file ijms-21-05953-s001.pdf]

Issue Number: 20206241314495_30314819

For Submission

Volunteer Certificate

Name :	[redacted]
Resident Registration Number :	690723-*******
Address :	[redacted]

- Attendance Days: 03/03/2020 ~ 05/19/2020
- Total Number of Hours: 71 Hours
- Description of Activities: Medical support volunteering related to COVID-19

This is to certify that (Jong-hoon Lee) has participated in volunteer activities as above.

June 24, 2020

Gangbuk-Gu Volunteer Center

강북구 자원봉사 센터인

Director : Hee-jung You (Phone : 82-02-901-6647)

◆ This is to certify the participation of the concerned person in volunteering as above. Any falsification or alteration of this certificate can be checked by entering the issue number in the certificate validation menu of the "1365 volunteer portal(www.1365.go.kr)." Validation of this issue number will be available within 90 days upon issuance.

* Description of volunteering issued from related agencies have been consolidated in link with the information from the portal.

Issue Number: 20206241314495_30314819 For Submission

■ Description of volunteer activities

Dates	Hours	Fields of activity	Description	Volunteer Agency
05/19/2020	6	Disaster and Emergency	Medical support volunteering related to COVID-19	Gangbuk-Gu Volunteer Center<br>Seoul Volunteer Center
05/18/2020	6	Disaster and Emergency	Medical support volunteering related to COVID-19	Gangbuk-Gu Volunteer Center<br>Seoul Volunteer Center
05/17/2020	5	Disaster and Emergency	Medical support volunteering related to COVID-19	Gangbuk-Gu Volunteer Center<br>Seoul Volunteer Center
05/16/2020	5	Disaster and Emergency	Medical support volunteering related to COVID-19	Gangbuk-Gu Volunteer Center<br>Seoul Volunteer Center
05/15/2020	6	Disaster and Emergency	Medical support volunteering related to COVID-19	Gangbuk-Gu Volunteer Center<br>Seoul Volunteer Center
05/14/2020	6	Disaster and Emergency	Medical support volunteering related to COVID-19	Gangbuk-Gu Volunteer Center<br>Seoul Volunteer Center
05/13/2020	6	Disaster and Emergency	Medical support volunteering related to COVID-19	Gangbuk-Gu Volunteer Center<br>seoul Volunteer Center
05/12/2020	6	Disaster and Emergency	Medical support volunteering related to COVID-19	Gangbuk-Gu Volunteer Center<br>Seoul Volunteer Center
03/14/2020	5	Disaster and Emergency	Medical support volunteering related to COVID-19	Gangbuk-Gu Volunteer Center<br>Seoul Volunteer Center
03/08/2020	6	Disaster and Emergency	Medical support volunteering related to COVID-19	Gangbuk-Gu Volunteer Center<br>Seoul Volunteer Center

Director : Hee-jung You (Phone : 82-02-901-6647)

◆ This is to certify the participation of the concerned person in volunteering as above. Any falsification or alteration of this certificate can be checked by entering the issue number in the certificate validation menu of the "1365 volunteer portal(www.1365.go.kr)." Validation of this issue number will be available within 90 days upon issuance.

* Description of volunteering issued from related agencies have been consolidated in link with the information from the portal.

Issue Number: 20206241314495_30314819 For Submission

■ Description of volunteer activities

03/07/2020	5	Disaster and Emergency	Medical support volunteering related to COVID−19	Gangbuk−Gu Volunteer Center<br>seoul Volunteer Center
03/04/2020	5	Disaster and Emergency	Medical support volunteering related to COVID−19	Gangbuk−Gu Volunteer Center<br>Seoul Volunteer Center
03/03/2020	5	Disaster and Emergency	Medical support volunteering related to COVID−19	Gangbuk−Gu Volunteer Center<br>Seoul Volunteer Center

Director : Hee−jung You (Phone : 82−02−901−6647)

◆ This is to certify the participation of the concerned person in volunteering as above. Any falsification or alteration of this certificate can be checked by entering the issue number in the certificate validation menu of the "1365 volunteer portal(www.1365.go.kr)." Validation of this issue number will be available within 90 days upon issuance.

* Description of volunteering issued from related agencies have been consolidated in link with the information from the portal.
